# LncRNA PWAR6 regulates proliferation and migration by epigenetically silencing YAP1 in tumorigenesis of pancreatic ductal adenocarcinoma

**DOI:** 10.1111/jcmm.16480

**Published:** 2021-04-08

**Authors:** Shanshan Huang, Yaqi Li, Jinhua Hu, Li Li, Zhen Liu, Hui Guo, Bailing Jiang, Jun Chen, Junhe Li, Xiaojun Xiang, Jun Deng, Jianping Xiong

**Affiliations:** ^1^ Department of Oncology The First Affiliated Hospital of Nanchang University Nanchang China; ^2^ Department of Oncology The Affiliated Xinyu Hospital Nanchang University Xinyu China

**Keywords:** migration, pancreatic ductal adenocarcinoma, proliferation, PWAR6, YAP1

## Abstract

Long non‐coding RNAs (lncRNAs) are a novel class of regulators in multiple cancer biological processes. However, the functions of lncRNAs in pancreatic ductal adenocarcinoma (PDAC) remain largely unknown. In this study, we identified PWAR6 as a frequently down‐regulated lncRNA in PDAC samples as well as a panel of pancreatic cancer cell lines. Down‐regulated PWAR6 was associated with multiple clinical outcomes, including advanced tumour stage, distant metastasis, and overall survival of PDAC patients. In our cell‐based assays, ectopic expression of PWAR6 dramatically repressed PDAC cells proliferation, invasion and migration, accelerated apoptosis, and induced cell cycle arrest at G0/G1 phase. In contrast, depletion of PWAR6 mediated by siRNA exhibited opposite effects on PDAC cell behaviours. In vivo study further validated the anti‐tumour role of PWAR6 in PDAC. By taking advantage of available online sources, we also identified YAP1 as a potential PWAR6 target gene. Negative correlation between YAP1 and PWAR6 expressions were observed in both online database and our PDAC samples. Notably, rescue experiments further indicated that YAP1 is an important downstream effector involved in PWAR6‐mediated functions. Mechanistically, PWAR6 could bind to methyltransferase EZH2, a core component of Polycomb Repressive Complex 2 (PRC2) in regulating gene expression, and scaffold EZH2 to the promoter region of YAP1, resulting in epigenetic repression of YAP1. In conclusion, our data manifest the vital roles of PWAR6 in PDAC tumorigenesis and underscore the potential of PWAR6 as a promising target for PDAC diagnosis and therapy.

## 1 INTRODUCTION

Pancreatic ductal adenocarcinoma (PDAC), a highly aggressive malignancy with limited efficacy of available therapies, accounts for the fourth leading cause of cancer‐related death worldwide.^1^, ^2^ Due to the lack of early diagnosis and its rapid progression, the 5‐year survival rate in PDAC is about 6%.^1^ Given the fact that PDAC is resistant to most therapies currently used in clinic, there is a strong demand to identify novel targets for development of new treatment options that could potentially improve clinical outcomes for PDAC patients.^3^ However, the complexity of its pathogenic lesions makes the study of the molecular mechanisms underlying PDAC very challenging.

Long non‐coding RNAs (lncRNAs) are a subclass of non‐coding transcripts with a length >200 nucleotides.^4^ While previously considered as noises of genetic materials, lncRNAs have now been greatly appreciated for its involvement in various biological processes, including transcriptional regulation, RNA processing, translational control, epigenetic modification, and posttranslational modification.^5^, ^6^, ^7^ In human cancers, especially in pancreatic cancer, aberrant lncRNA expression in PDAC indicates its vital roles in tumorigenesis.^8^ Indeed, various lncRNAs have been reported to participate in the development, progression, metastasis, and chemotherapy‐resistance of pancreatic cancer.^9^, ^10^, ^11^ For example, elevated expression of lncRNA‐BX111 in pancreatic cancer tissues is associated with shorter overall survival time of patients. By activating the transcription of ZEB1 through recruiting transcriptional factor Y‐box protein (YB1) to its promoter region, lncRNA‐BX111 promotes the epithelia‐mesenchymal transition (EMT) in pancreatic cancer cells.^9^ Research from Li et al^10^ suggested that lncRNA NORAD acts as a ceRNA to regulate the expression of the small GTP binding protein RhoA through competition for hsa‐miR‐125a‐3p, and thus promotes progression of pancreatic tumour. In addition, some tumour‐specific expressing lncRNAs are ideal and excellent targets for designing the novel therapeutic strategies against human malignancies^12^

In the present study, we investigated the clinical significance and biological functions of a poorly understood lncRNA in PDAC. Prader Willi/Angelman Region RNA 6 (PWAR6) is a 4618‐bp non‐conserved lncRNA located on chromosome 15 in humans and contains one exon. The non‐coding nature of lncRNA PWAR6 was confirmed by coding‐potential analysis (Figure S1). PWAR6 was identified as a protective lncRNA in glioma as it exhibits grade‐specific dynamic expression and regulates hallmark‐related genes,^13^ but its characteristic in PDAC remains largely elusive. Here, we assessed the expression patterns of PWAR6 in PDAC tissues and cell lines as well as its correlations with clinical outcomes in PDAC. In addition, we determined the biological functions of PWAR6 in PDAC via conducting a series of cell‐based experiments. Finally, we explored the molecular mechanism of PWAR6 in PDAC cell proliferation and metastasis, providing novel insight in determining PWAR6 as a potential therapeutic and prognostic target in PDAC.

## 2 MATERIALS AND METHODS

### 2.1 Clinical samples

Primary pancreatic ductal adenocarcinoma tissues and their paired adjacent benign pancreatic tissues were obtained from 63 patients who underwent surgery resection at the First Affiliated Hospital of Nanchang University (Nanchang, Jiangxi, PR China). All specimens were immediately frozen in liquid nitrogen after surgery and stored at –80°C for future use. This study was approved by the Ethics Committee of the First affiliated hospital of Nanchang University. Written informed consent was obtained from all patients. Clinicopathological characteristics of all patients were shown in Table 1.

**TABLE 1:** Relationship between PWAR6 expression and clinicopathological variables (n = 63)

Variables	Number	PWAR6 expression		*P* value
		High (n = 32)	Low (n = 31)	
Gender				
Male	35	16	19	0.367
Female	28	16	12	
Age^a^				
>64	33	20	13	0.102
<64	30	12	18	
Node metastasis				
Yes	44	19	25	0.066
No	19	13	6	
Distant metastasis				
Yes	29	8	21	**0.001**
No	34	24	10	
Tumour size				
<2 cm	31	18	13	0.256
>2 cm	32	14	18	
Differentiation				
Low/undifferentiated	37	15	22	0.052
High/moderate	26	17	9	
TNM stage				
I/II	33	21	12	**0.032**
III/IV	30	11	19	

^a^ Age = mean age.

A *P* value < 0.05 was considered statistically significant. The values less than 0.05 were bolded.

### 2.2 Cell culture

Human pancreatic ductal adenocarcinoma cell lines (Capan‐1, AsPC‐1, SW1990, BxPC‐3, PANC‐1) and one human pancreatic duct epithelial cell line (HPDE6‐C7) were obtained from the Cell Bank of the Chinese Science Academy (Shanghai, China). HPDE6‐C7 cells were cultured in keratinocyte serum‐free medium (Thermo Fisher Scientific). BxPC‐3 cells were maintained in RPMI‐1640 medium (Hyclone). Capan‐1, AsPC‐1, SW1990 and PANC‐1 were cultured in Dulbecco's modified Eagle's medium (Hyclone). Both DMEM and RPMI1640 were supplemented with 10% FBS (Gibco; Thermo Fisher Scientific), 100 U/mL streptomycin and 100 U/mL penicillin. All cells were cultured at 37°C in a humidified atmosphere with 5% CO_2_.

### 2.3 Cell transfection

Scramble siRNA, siRNAs for PWAR6 and expression vectors for YAP1 were synthesized and obtained from Genepharma. The siRNAs and plasmid were transfected into PDAC cells using Lipofectamine2000 (Invitrogen) according to the manufacturer's protocols. 48 hours after transfections, cells were harvested for qRT‐PCR or western blot analysis. The sequences for siRNAs are as follows: YAP1 siRNA, 5′‐CUGCCACCAAGCUAGAUAATT‐3′. PWAR6 siRNA#1, 5′‐GGAAAUUCCUUUCCUCCAATTUUGGAGGAAAGGAAUUUCCTT‐3′; PWAR6 siRNA#2, 5′‐GGAGUUACCUCCAUGUGAATTUUCACAUGGAGGUAACUCCTT‐3′; scramble siRNA, 5′‐UUCUCCGAACGUGUCACGUTT‐3′.

### 2.4 Cell proliferation analysis

Cell viability was determined by using CCK‐8 kit (Dojindo Molecular Technologies) as per the manufacturer's instruction. For colony formation assay, the transfected cells were seeded into 6‐well plates and maintained in proper medium containing 10% FBS for 10 days. Colonies were then fixed with methanol and stained with 0.1% crystal violet (Sigma‐Aldrich) in PBS for 15 minutes. Only colonies that reached more than 50 cells were counted. BrdU experiment was performed by using a BrdU Cell Proliferation Assay Kit (Millipore) according to the manufacturer's instructions. The experiments were performed in triplicate and repeated at least three times.

### 2.5 Flow cytometry analysis

For cell cycle distribution analysis, transfected cells were collected and fixed with 70% ethanol at 4°C overnight. The cells were then resuspended in cold PBS and incubated with 200 μg/mL of RNase at 37°C for 30 minutes, followed by labelling with 50 μg/mL propidium iodide (PI) (BD Biosciences) and flow cytometry analysis. The percentages of cells in G0‐G1, S, and G2‐M phases were counted and compared. For cell apoptosis assay, cells were dual stained with 2.5 μg/mL of Annexin V and 50 μg/mL of PI (BD Biosciences) for 15 minutes at room temperature in the dark. Cells were analysed using a BD FACSCanto II system (BD Biosciences). The experiments were performed in triplicate and repeated at least three times.

### 2.6 Cell invasion and migration assay

Transfected cells were plated onto the upper insertion chamber (Millipore), that was either coated (to assess invasion) or non‐coated (to assess migration) with 100 µL matrigel (BD Biosciences), in serum‐free media. While the lower chamber was supplemented with culture medium containing 10% FBS. After 24 hours incubation, the cells remaining on the upper membrane were removed with cotton wool. Cells on the bottom of the filter were fixed with methanol, stained with 0.1% crystal violet, imaged and counted using an IX71 inverted microscope (Olympus). The experiments were performed in triplicate and repeated at least three times.

### 2.7 Mouse xenograft experiments

The animal protocol of this study was approved by the Institutional Animal Care and Use Committee (IACUC) of The First Affiliated Hospital to Nanchang University (Nanchang, China). Seven‐week‐old female NOD/SCID mice were randomized into two groups (five mice in each). To assess the effect of sh‐PWAR6 on PDAC tumorigenesis, 5 × 10^6^ BxPC‐3 cells infected with sh‐LINC01133 or sh‐scramble were injected into the right flanks of NOD/SCID mice. Four weeks later, the mice were sacrificed and the tumours were harvested and weighed.

### 2.8 Western blot

Briefly, cell lysates were prepared in RIPA buffer, separated by 8% SDS‐PAGE and then transferred to PVDF membranes (Millipore). Membrane was blocked with 5% non‐fat milk in TBS at 4°C overnight and then incubated with specific antibodies at room temperature for 1 hour, followed by incubation with HRP‐conjugated secondary antibody at room temperature for another 1 hour. The bands were visualized using electrochemiluminescent (ECL) detection system (Thermo Fisher Scientific). Anti‐EZH2 and anti‐GAPDH antibodies were purchased from Abcam, anti‐YAP1, anti‐caspase‐3, anti‐BAX and anti‐Bcl‐2 antibodies were obtained from Cell Signaling Technology (Massachusetts).

### 2.9 Quantitative reverse transcription PCR

Total RNA from tissues or cell lines were extracted by using Trizol reagent (Invitrogen) according to the manufacturer's instructions. RNA was then reverse‐transcribed to cDNA using an miScript SYBR Green PCR kit (Invitrogen). SYBR Green PCR Master Mix (Applied Biosystems) was used to quantify the mRNA levels of target genes. qRT‐PCR was performed on an ABI7500 system (Applied Biosystems) and the conditions were as follows: an initial temperature of 95°C for 2 min and 40 cycles of 95°C for 10 seconds, 60°C for 10 seconds, and 72°C for 40 seconds. The primer sequences were PWAR6, 5ʹ‐CTGTGCCGTTTGGCATAAGA‐3ʹ (forward) and 5ʹ‐TCACCACCTCACAGATCACC‐3ʹ (reverse). U6, 5ʹ‐GCTT CGGCAGCAGCACATATACTAAAAT‐3ʹ (forward) and 5ʹ‐CGCTT CACGAATTTGCGTGTCAT‐3ʹ (reverse). YAP1: 5′‐CAGGAGCCCTGACTCCACAG‐3′ (forward) and 5′‐TTGCCATCTCCCAACCTGCT‐3′ (reverse); GAPDH: 5′‐CAGGGCTGCTTTTAACTCTGGT‐3′ (forward) and 5′‐GATTTTGGAGGGATCTCGCT‐3′ (reverse). The relative mRNA expression levels were calculated using the $2^{‐\Delta \Delta C_{T}}$ method.

### 2.10 Subcellular fractionation

The Cytoplasmic and Nuclear RNA Purification Kit (Norgen) was used to separate the nuclear and cytoplasmic fractions according to the manufacturer's instructions.

### 2.11 RNA immunoprecipitation

RNA immunoprecipitation (RIP) experiments were performed using a Magna RIP RNA‐Binding Protein Immunoprecipitation Kit (Millipore), according to the manufacturer's instructions. Antibodies for RIP assays of EZH2 and SUZ12 were from Abcam. IgG was used as a negative control and obtained from Santa Cruz Biotechnology.

### 2.12 RNA pull‐down assay

Biotin‐labelled PWAR6 was obtained by T7 RNA polymerase (Promega) and biotin RNA tagged mixtures (Roche). Cell lysates were prepared in RIP buffer and then mixed with biotin‐labelled PWAR6 RNAs. After incubation at 4°C for 1 hour, the streptavidin‐coated beads (Thermo Fisher Scientific) were added in the reaction. The precipitated proteins were separated by SDS‐PAGE and detected by western blot analysis.

### 2.13 Chromatin immunoprecipitation (ChIP) assay

The ChIP assay was carried out using the EZ ChIP™ Chromatin Immunoprecipitation Kit (Millipore) according to the manufacturer's instructions. Briefly, post transfected cells were subjected to chromatin cross‐linking, sonication and then immunoprecipitation of DNA‐protein complex using anti‐EZH2 (Abcam) or anti‐H3K27me3 (Millipore) antibody according to the kit's protocol. Standard qRT‐PCR was performed to quantify the immunoprecipitated DNA.

### 2.14 Statistical analysis

Statistical analysis was performed using SPSS 22.0. The relationship between the expression level of PWAR6 and clinicopathological characteristics of PDAC patients was analysed by chi‐square test. OS rates were calculated by Kaplan‐Meier method and compared using the log‐rank test. Pearson correlation analysis was applied to assess the correlation between PWAR6 and YAP1. Student's *t* test or one‐way ANOVA analysis was used for calculating intergroup differences. A *P* value <0.05 was considered statistically significant.

## 3 RESULTS

### 3.1 LncRNA PWAR6 is down‐regulated in human PDAC and correlates with poor prognosis

**FIGURE 1:**
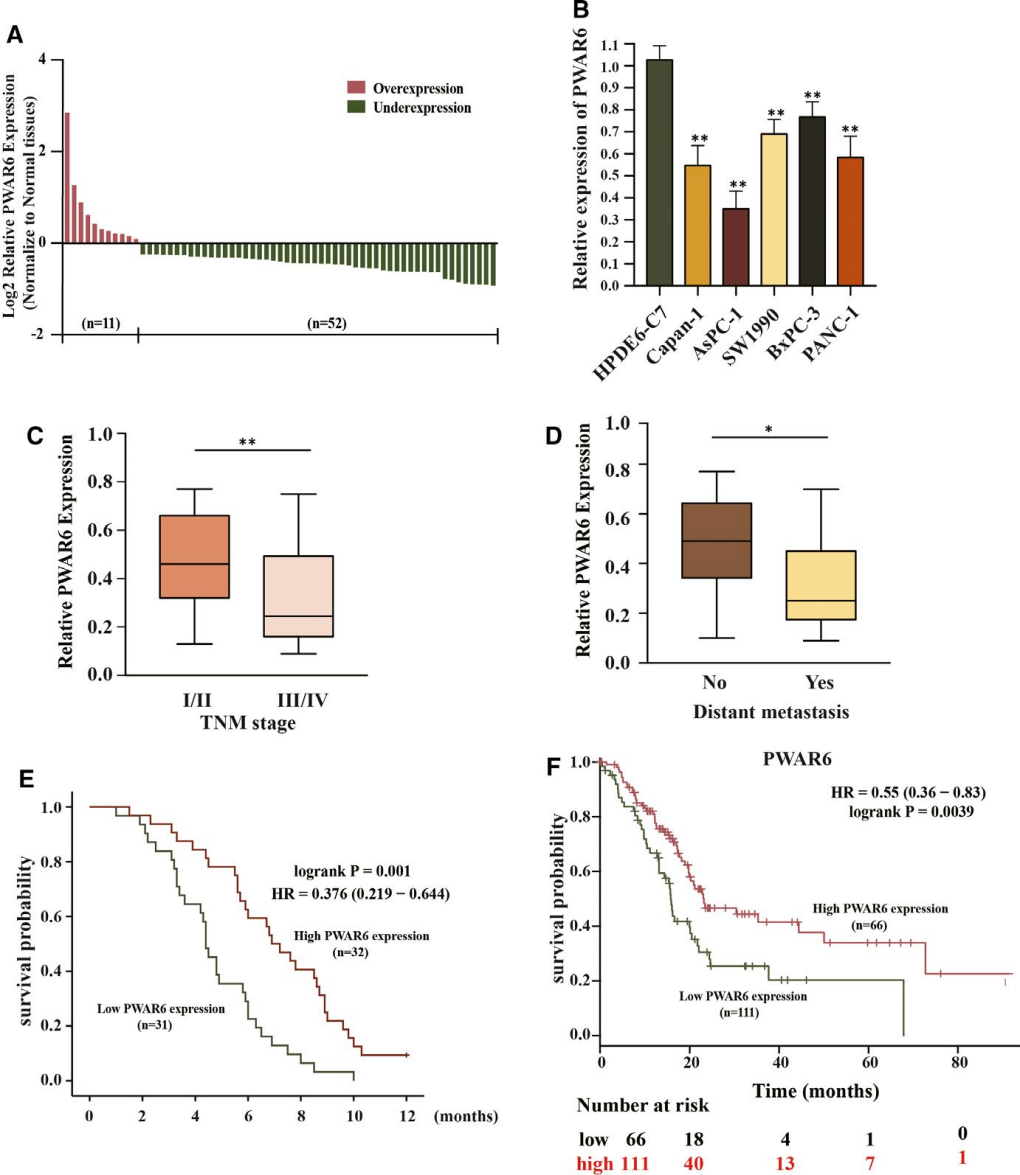
LncRNA PWAR6 is down‐regulated in human PDAC and correlates with poor prognosis. A, PWAR6 was detected in 63 pairs of pancreatic ductal adenocarcinoma (PDAC) tissues and adjacent benign pancreatic tissues by qRT‐PCR. The expression of PWAR6 was found to be frequently down‐regulated in PDAC tissues as compared with that in adjacent benign pancreatic tissues. B, The relative expression of PWAR6 was assessed in PDAC cell lines (Capan‐1, AsPC‐1, SW1990, BxPC‐1, and PANC‐1) and a normal human pancreatic ductal epithelial cell line (HPDE6). C, D, PWAR6 expression was significantly lower in patients with advanced TNM stage (C) and tumour distant metastasis (D). E, F, (E) Patients with high PWAR6 expression level manifested longer survival time (*P* = 0.001, log‐rank test), and data extracted from Kaplan‐Meier website further confirmed our result (F)

The mRNA expression level of PWAR6 was evaluated in 63 PDAC samples and their paired benign pancreatic tissues by qRT‐PCR. As shown in Figure 1A, down‐regulation of PWAR6 expression was clearly observed in 82.5% (52 of 63 paired) PDAC as compared with benign tissues. This down‐regulation was further supported by the finding that the mRNA expression levels of PWAR6 in 5 PDAC cell lines (Capan‐1, AsPC‐1, SW1990, BxPC‐1, and PANC‐1) were all significantly lower than that in normal human pancreatic ductal epithelial cell line HPDE6 (Figure 1B). Next, we further explored the correlation between PWAR6 expression and clinical characteristics of patients with PDAC. We found that patients with advanced tumour stage and distant metastasis tend to have lower PWAR6 expression than patients with early tumour stage and without distant metastasis (0.3183 ± 0.19983 versus 0.33 ± 0.19284, *P* = 0.0035 and 0.3253 ± 0.19155 versus 0.4585 ± 0.20593, *P* = 0.0105), respectively (Figure 1C,D). We then divided PDAC samples into PWAR6 high group (n = 32) and PWAR6 low group (n = 31) based on the median expression level of PWAR6 for Chi‐square test to evaluate clinic‐parameters between the two groups. The expression level of PWAR6 was significantly associated with tumour stage (*P* = 0.032) and distant metastasis status (*P* = 0.001). Other clinic factors such as patients’ gender and age, node metastasis, tumour size, or histological grade weren't associated with PWAR6 expression in this study (Table 1). We next analysed the prognostic role of PWAR6 in the same cohort of PDAC patients. Kaplan‐Meier and log‐rank test were used to evaluate the expression of PWAR6 on the clinic outcome of PDAC patients. Patients with higher PWAR6 expression manifested longer overall survival than patients with lower PWAR6 expression (7.1438 ± 2.70896 months versus 4.7645 ± 2.07839 months, *P* = 0.001) (Figure 1E). In addition, the data retrieved from KM‐Plotter database (http://kmplot.com/analysis/) further confirmed the prognostic role of PWAR6 in PDAC patients (Figure 1F). Taken together, these results suggest that PWAR6 is frequently down‐regulated in PDAC and associated with advanced tumour stage, distant metastasis status and poor prognosis, indicating its potential tumour suppressor role in PDAC.

### 3.2 LncRNA PWAR6 regulates PDAC cell progression both in vitro and in vivo

**FIGURE 2:**
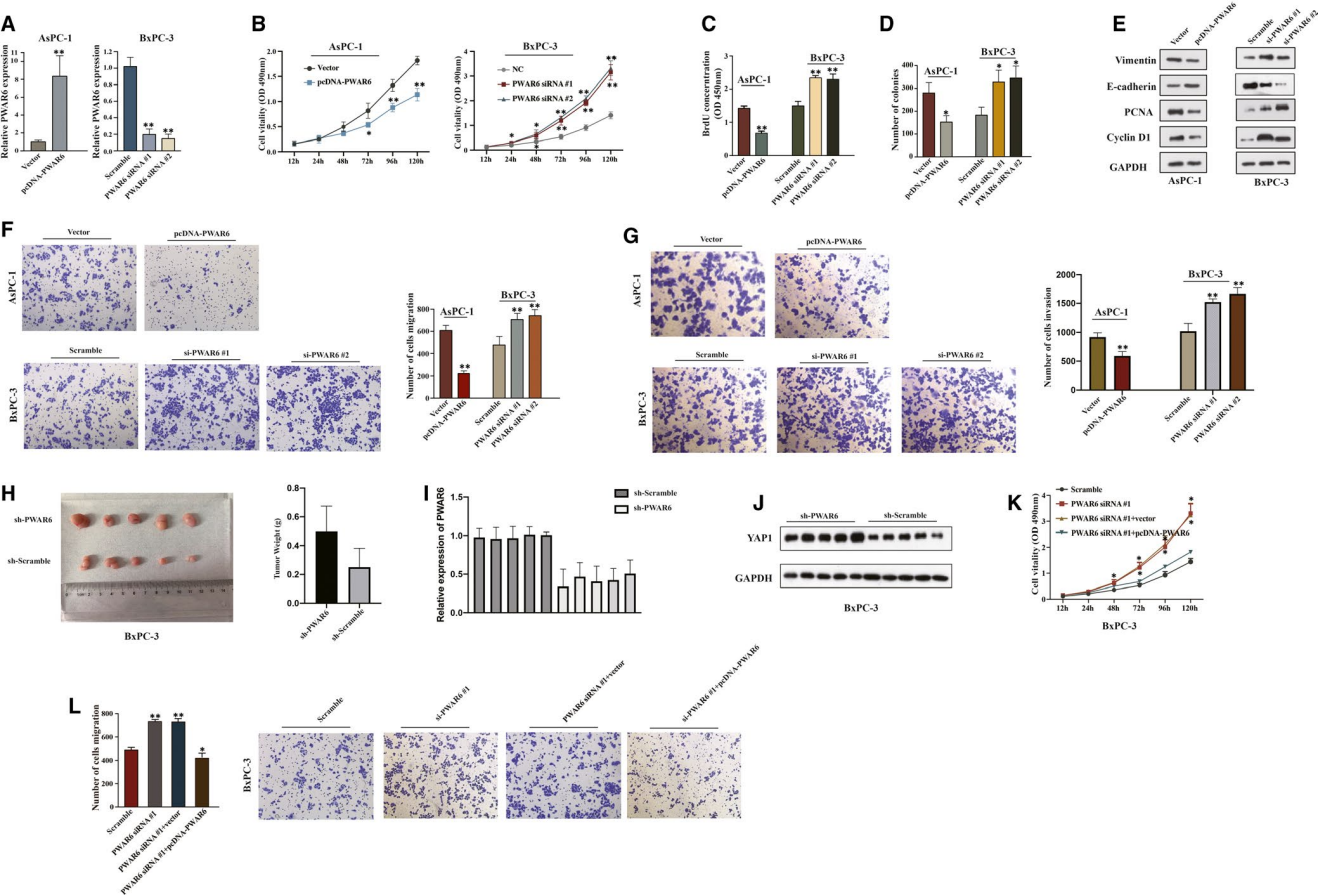
LncRNA PWAR6 regulates PDAC cell proliferation, invasion and migration in vitro and in vivo. A, PWAR6 expression was exogenously manipulated using PWAR6 siRNAs or PWAR6 cDNA in AsPC‐1 and BxPC‐3 cells, respectively and assessed by using qRT‐PCR. B‐D, The cell proliferation ability of AsPC‐1 and BxPC‐3 cells after transfecting with PWAR6 siRNAs or overexpression plasmid was determined by CCK‐8 assay (B), BrdU assay (C) and colony formation assay (D). E, Cells were transfected with PWAR6 siRNAs, plasmid or the negative control, and then subjected to Western blot to evaluate the expression of E‐cadherin, vimentin, PCNA and cyclin D1. F‐G, The cell migration ability of AsPC‐1 and BxPC‐3 cells after transfecting with PWAR6 siRNAs or overexpression plasmid was determined by Transwell migration assay (F). F, The cell invasion ability of AsPC‐1 and BxPC‐3 cells after transfecting with PWAR6 siRNAs or overexpression plasmid was determined by Transwell invasion assay (G). H, Xenograft tumours were harvested at the end of experiment and weighted. I‐J, The levels of PWAR6 and YAP1 were detected in ten tumour samples by qRT‐PCR (I) and WB analysis (J). K‐L, The cell proliferation and migration ability of BxPC‐3 cells transfected with PWAR6 siRNA was tested after co‐transfecting with pcDNA‐PWAR6 by conducting CCK‐8 assay (K) and Transwell migration assay (L)

To investigate whether PWAR6 is involved in the tumorigenesis of PDAC, the plasmid‐mediated overexpression and siRNA‐mediated knockdown were applied to manipulate the expression of PWAR6 in PDAC cell lines. According to the basal PWAR6 expression level in different PDAC cells, we then selected PDAC ASPC‐1 cells with relatively low PWAR6 expression for overexpression of PWAR6 and BxPC‐3 cells with relatively high PWAR6 expression to knockdown of PWAR6 expression (Figure 2A). The results from CCK‐8 assay revealed that overexpression of PWAR6 dramatically decreased cell viability of AsPC‐1 cells as compared to vector control. In contrast, knockdown of PWAR6 by two sets of siRNAs increased the viability of BxPC‐3 cells (Figure 2B). Consistently, BrdU assay and colony formation assay showed that ectopic expression of PWAR6 significantly repressed the proliferation rate and the clonogenic survival of AsPC‐1 cells, while PWAR6 knockdown clearly showed opposite effects on BxPC‐3 cells, as presented in Figure 2C,D. We also checked the levels of cell proliferation markers (PCNA and cyclin D1) and invasion markers (E‐cadherin and Vimentin) by performing western blot assay. PWAR6‐overexpressing ASPC‐1 cells showed a higher expression level of E‐cadherin, but a lower expression level of vimentin, PCNA and cyclin D1 than control cells. In contrast, knockdown of PWAR6 expression had the opposite results in BxPC‐3 cells (Figure 2E). In line with these observations, our transwell invasion and migration assays showed that overexpression of PWAR6 inhibited the ability of AsPC‐1 cell to invasion and migration, while knockdown of PWAR6 promoted BxPC‐3 cell invasion and migration when compared with control cells (Figure 2F,G). In order to investigate the role of PWAR6 on PDAC tumorigenesis in vivo, BxPC‐3 cells transferred sh‐PWAR6 or sh‐Scramble were injected into NOD/SCID mice, and monitored tumour size for 28 days. As illustrated in Figure 2H, PWAR6 knockdown markedly promoted the growth of xenograft tumours, suggesting that PWAR6 might possess a tumour suppressor role in PDAC. In addition, we performed WB analysis using the xenograft tumours, and found that the protein levels of YAP1 were up‐regulated in shPWAR6 group (Figure 2I,J). This result indicated that PWAR6 depletion promotes tumour growth by activating YAP1. To rule out the possibility that these effects were caused non‐specifically, we re‐introduced PWAR6 into cells that were already transfected with PWAR6 siRNAs, and the results shown in Figure 2K,L clearly demonstrated that in addition to reproduced effects of PWAR6 knockdown on BxPC‐3 cell viability and migration, these effects were successfully rescued by PWAR6 overexpression. Collectively, these findings confirmed the tumour suppressor role of PWAR6 in PDAC cells.

### 3.3 Overexpression of LncRNA PWAR6 inhibits cell cycle and promotes cell apoptosis

**FIGURE 3:**
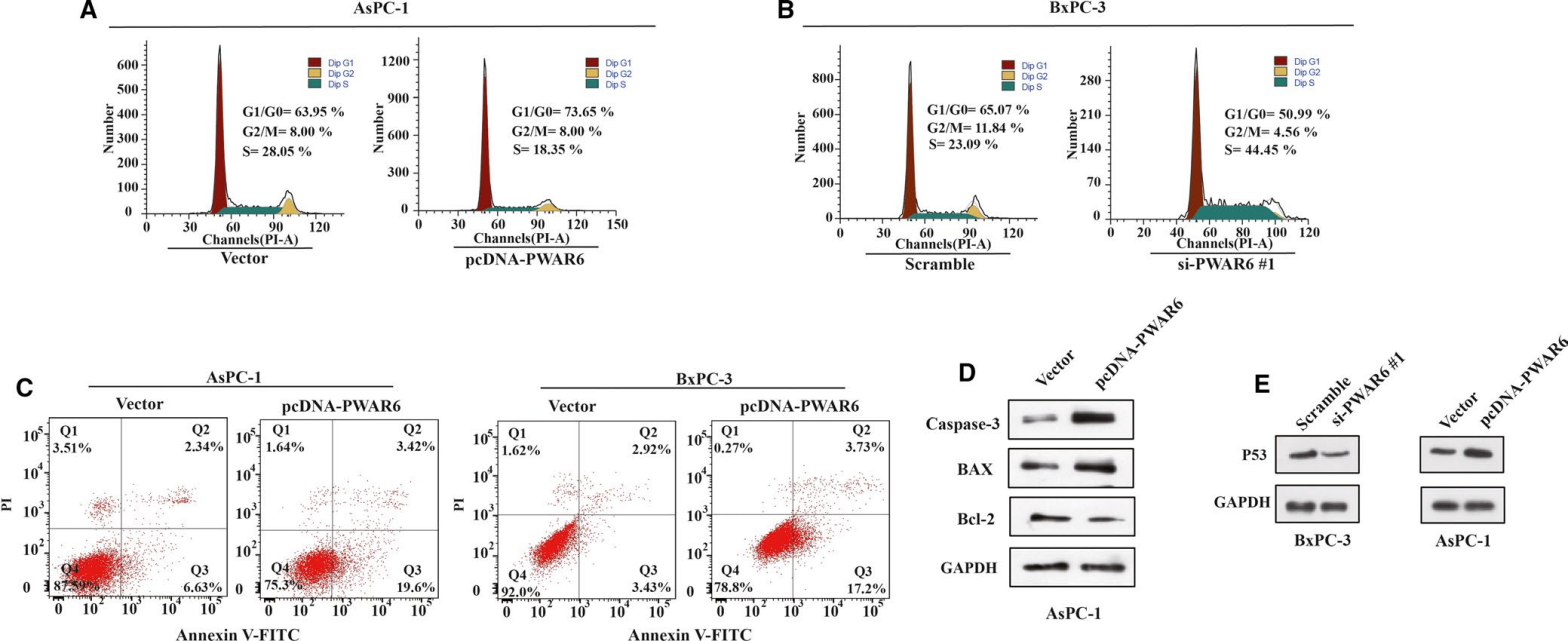
Overexpression of LncRNA inhibits cell cycle and promotes cell apoptosis. A‐B, The effect of PWAR6 up‐regulation (A) and down‐regulation (B) on cell cycle progression of PDAC cells was detected by flow cytometry analysis. C, The apoptotic rates of pcDNA‐PWAR6 transfected PDAC cells were measured by flow cytometry. LL dead cells, UL viable cells, LR early apoptotic cells, UR terminal apoptotic cells. D, AsPC‐1 cells were grown and subjected to pcDNA‐PWAR6 plasmid transfection, the protein levels of caspase‐3, Bcl‐2 and BAX were accessed by Western blot. E, AsPC‐1 cells and BxPC‐3 cells were grown and subjected to pcDNA‐PWAR6 plasmid or PWAR6 siRNA transfection, the protein levels of p53 was accessed by Western blot

The above data indicate that PWAR6 regulates cell proliferation and migration in PDAC, and these results promoted us to further investigate whether PWAR6 could influence cell cycle and cell apoptosis. As expected, flow cytometry analysis revealed that PWAR6 overexpression led to a significant accumulation of population in G1/G0 phase and a decrease in the percentage of cells in S phase (Figure 3A). On the contrary, in response to PWAR6 knockdown, the cell population was skewed towards S phase and the percentage of cells in G1/G0 phase decreased (Figure 3B). The cell apoptosis was analysed by flow cytometry using Annexin V FITC/PI staining in AsPC‐1 and BxPC‐3 cells as shown in Figure 3C. Cells transfected with PWAR6 plasmid showed significantly increased percentage of apoptotic cells, which is 23.02% as compared to 8.97% in control cells for AsPC‐1 and 20.93% as compared to 6.35% in control cells for BxPC‐3. In addition, the expression levels of apoptosis‐related proteins, such as caspase‐3, BAX and Bcl‐2 were also tested in PWAR6 overexpressed AsPC‐1 cells. As indicated in Figure 3D, PWAR6 up‐regulation increased the expression of caspase‐3 and BAX, while decreased the expression of Bcl‐2. Besides, the regulation of PWAR6 can also affect the level of a well‐documented tumour suppressor P53. We found that overexpression of PWAR6 elevates p53 protein level, while knockdown of PWAR6 inhibits p53 protein level (Figure 3E). Together, all these results demonstrated that PWAR6 overexpression inhibits cell cycle and promotes cell apoptosis.

### 3.4 LncRNA PWAR6 is responsible for the epigenetic repression of YAP1 by interacting with PRC2

**FIGURE 4:**
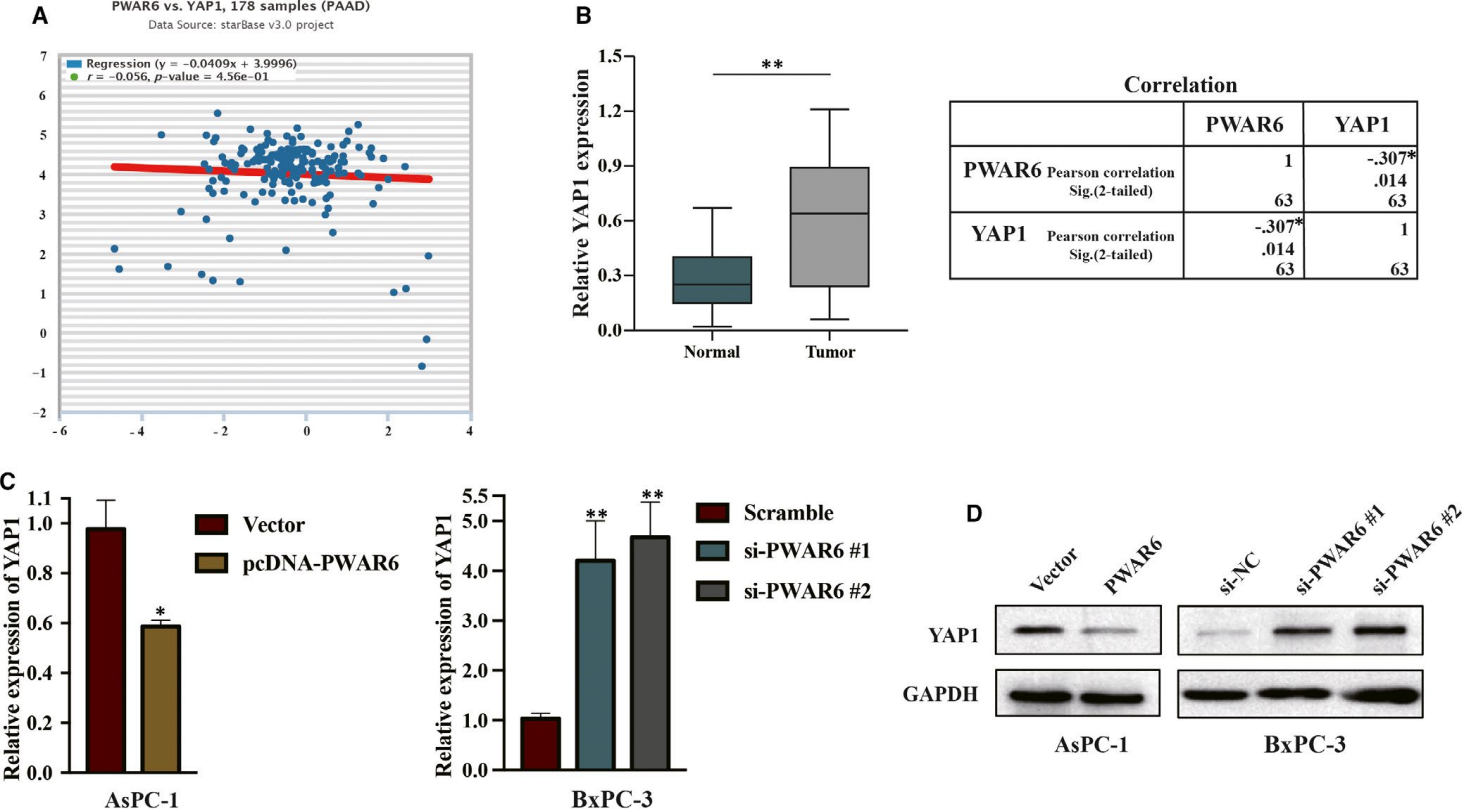
The expression of PWAR6 and YAP1 were negatively associated. A, Bioinformatical analysis of PWAR6 and YAP1 expression using the starBase v3.0 Pan‐Cancer Analysis and Networks Platform data. B, The expression level of YAP1 was significantly up‐regulated in PDAC tissues and was negatively correlated with PWAR6 expression level. C‐D, AsPC‐1 and BxPC‐3 cells were grown and subjected to PWAR6 siRNA or pcDNA‐PWAR6 plasmid transfection, the mRNA and protein levels of YAP1 in these cells were then accessed by qRT‐PCR (C) or Western blot (D)

We next sought to further explore the molecular mechanisms underlying PWAR6 functions. We first searched for potential PWAR6 target genes by employing starBase v3.0 Pan‐Cancer Analysis and Networks Platform (http://starbase.sysu.edu.cn/). Interestingly, the result in Figure 4A showed that PWAR6 exhibited a weak negative correlation trend with YAP1, a main downstream effector of Hippo pathway. Dysregulation of YAP1 affects transcription of various genes related to cell proliferation and migration, subsequently contributing to cancer development.^14^ This finding led us to evaluate the expression of YAP1 in the 63 paired PDAC samples mentioned above, and we found the expression of YAP1 was markedly increased in PDAC samples when compared with adjacent normal pancreatic tissues (Figure 4B, left panel). Pearson analysis indicated that the expressions of PWAR6 and YAP1 were significantly negatively correlated (Figure 4B, right panel). In line with this observation, our qRT‐PCR and western blot analysis showed that overexpression of PWAR6 in AsPC‐1 cells significantly reduced both mRNA and protein levels of YAP1, and siRNA knockdown of PWAR6 in BxPC‐3 cells up‐regulated YAP1 expression (Figure 4C,D).

**FIGURE 5:**
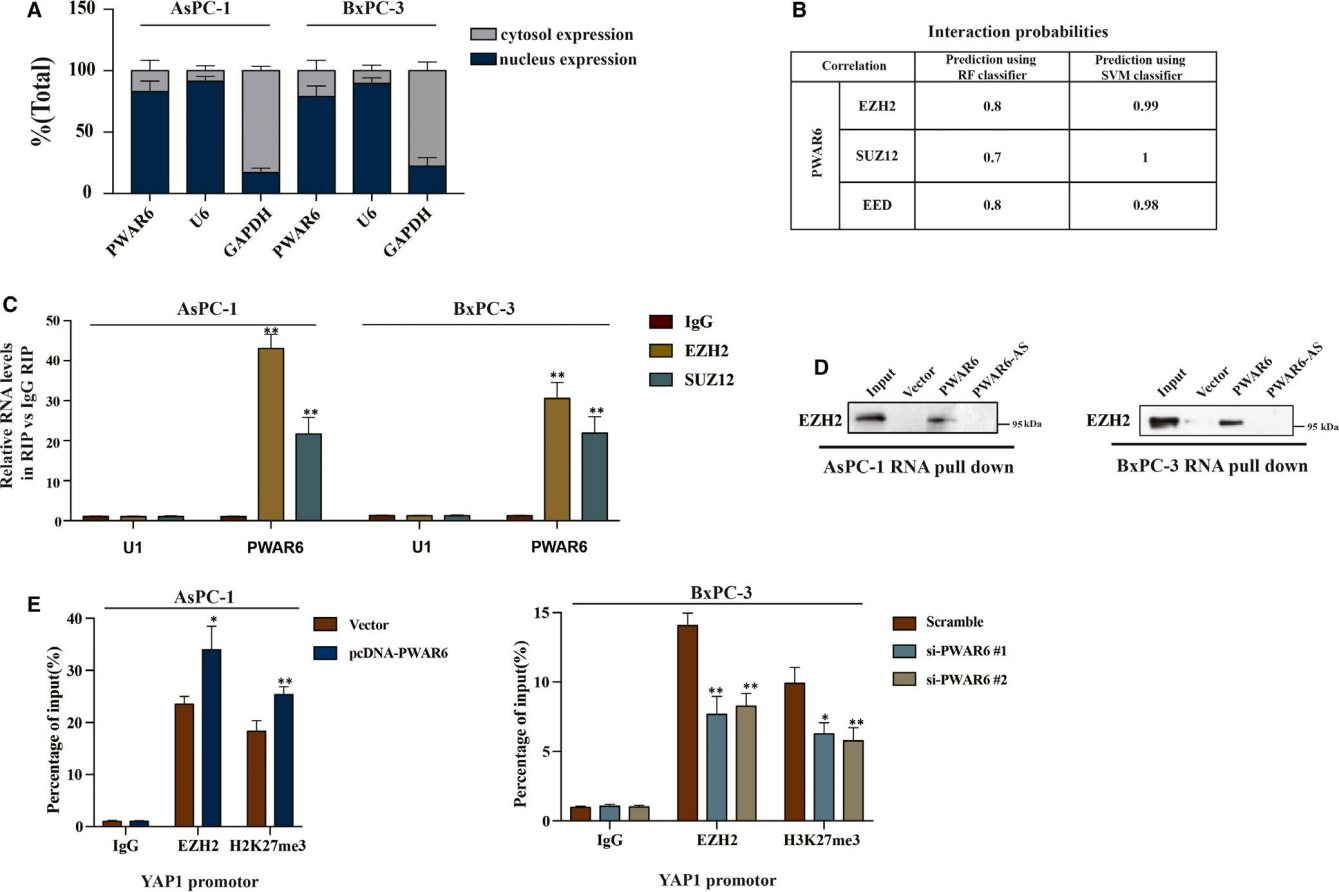
LncRNA is responsible for the epigenetic repression of YAP1 by interacting with PRC2. A, The PWAR6 subcellular location was determined in AsPC‐1 and BxPC‐3 cells. U6 was used as a nuclear marker and GADPH was used as a cytoplasmic marker. B, The binding probability between PWAR6 and PRC2 complex (SUZ12, EZH2, EED) was predicted in RNA‐protein interaction prediction website (RF and SVM scores >0.5). C, RIP assay validated the interaction between PWAR6 and EZH2/SUZ12 in PDAC cells. D, RNA pull‐down assay demonstrated that PWAR6 could specially retrieved EZH2 in PDAC cells. E, The results of ChIP assay shown that PWAR6 overexpression could dramatically enhancing the binding ability of EZH2 to YAP1 promoter region and promoting the methylation of H3K27me3 in PDAC cells, while PWAR6 down‐regulation contributed to the contrary results

According to previous reports, numerous lncRNAs could bind to chromatin‐modifying enzymes to promote epigenetic activation or silencing of its target gene expression.^15^ In order to better understand detailed mechanism of PWAR6 functions, we then examined the location of PWAR6 inside a cell. The results shown in Figure 5A demonstrated that PWAR6 was primarily located in the nucleus, where potential regulation on transcription mediated by PWAR6 could occur. Polycomb Repressive Complex 2 (PRC2), a multimeric enzymatic complex composed of EZH2, SUZ12 and EED, has histone methyltransferase activity and primarily trimethylates histone H3 on lysine 27.^16^ About 20% lncRNAs have been suggested to associate with PRC2 physically.^17^ Therefore, we hypothesized that PWAR6 could regulate its downstream target gene through binding to PRC2. To test this hypothesis, we performed RNA‐protein interaction prediction (http://pridb.gdcb.iastate.edu/RPISeq/references.php) and found a high possibility for PWAR6 to interact with EZH2, SUZ12 and EED (Figure 5B). Next, RNA‐RIP assay was carried out to verify the interaction between PWAR6 and PRC2. As shown in Figure 5C, PWAR6 could be specifically pulled down by EZH2 and SUZ12 antibodies, but not IgG negative control. In addition, RNA pull‐down assay demonstrated that PWAR6, rather than the vector control and PWAR6 antisense, could specifically retrieve EZH2 in PDAC cells (Figure 5D). We speculated that PWAR6 might epigenetically inhibit the expression of YAP1 by scaffolding EZH2. Our ChIP assay followed by qPCR analysis indicated that PWAR6 overexpression significantly enhanced EZH2 binding to and H3K27me3 levels on the YAP1 promoter region (Figure 5E, left panel). In contrast, the binding of EZH2 and the H3K27me3 levels were decreased in the promoter region of YAP1 when PWAR6 was knocked down (Figure 5E, right panel). Taken together, our data clearly indicate that PWAR6 binds to EZH2 and facilitates EZH2/PRC2‐mediated epigenetic modification of YAP1 promoter for suppression.

### 3.5 LncRNA PWAR6 promotes cancer progression via negatively regulating YAP1

**FIGURE 6:**
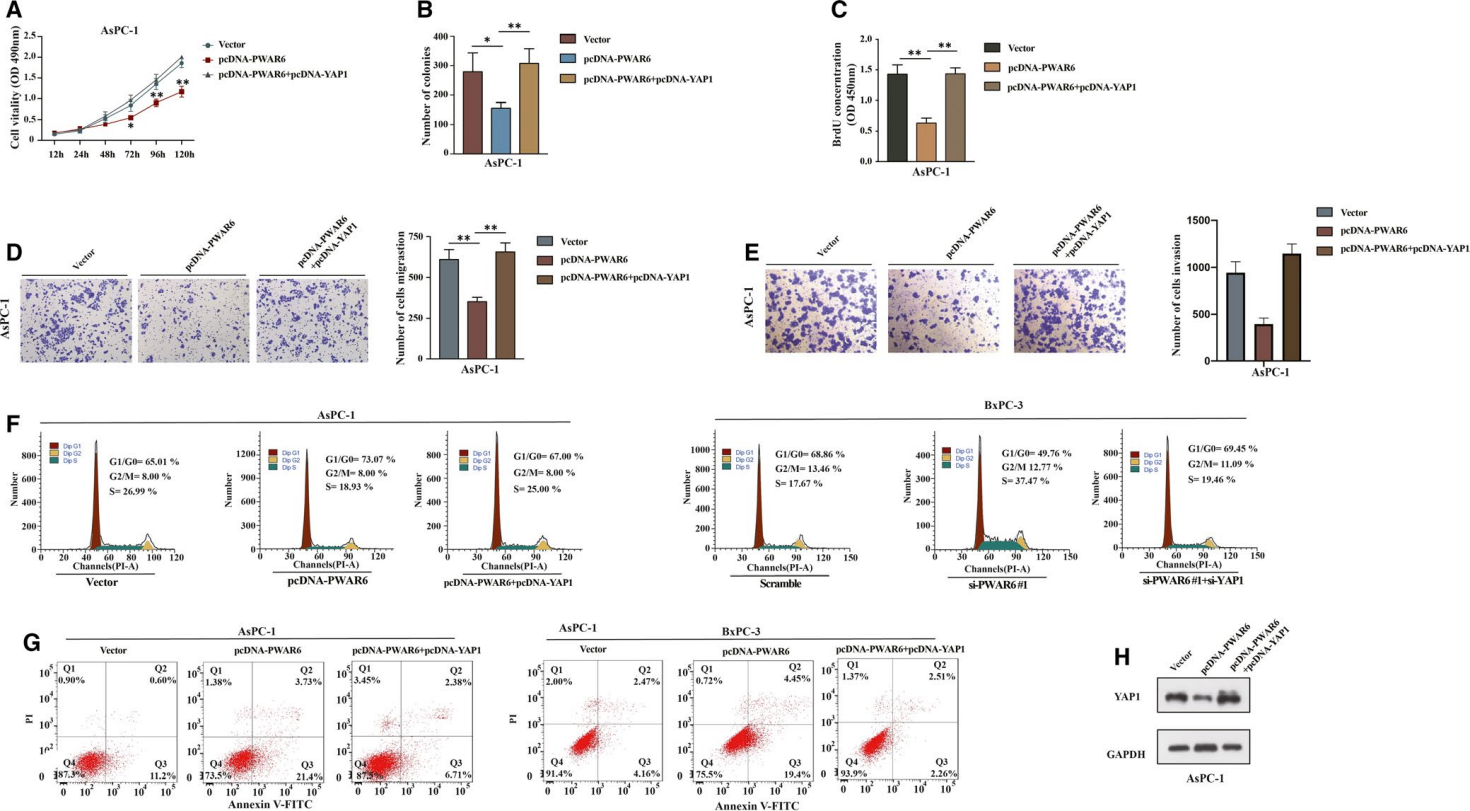
LncRNA promotes cancer progression via negatively regulating YAP1. A‐F, The cell proliferation, invasion and migration ability of AsPC‐1 cells transfected with PWAR6 siRNA was tested after co‐transfecting with pcDNA‐YAP1 by conducting CCK‐8 assay (A), colony formation assay (B), BrdU assay (C), Transwell migration assay (D) and Transwell invasion assay (E). F, AsPC‐1 cells were grown and co‐transfected pcDNA‐YAP1 and pcDNA‐PWAR6 or their negative controls and then subjected to flow cytometry analysis. BxPC‐3 cells were grown and co‐transfected YAP1 siRNA and PWAR6 siRNA#1 or their negative controls and then subjected to flow cytometry analysis. G, The apoptotic rates of pcDNA‐YAP1 and pcDNA‐PWAR6 or their negative controls co‐transfected PDAC cells were measured by flow cytometry. H, The expression level of YAP1 was measured by western blotting analysis in pcDNA‐PWAR6, pcDNA‐PWAR6 + pcDNA‐YAP1 or their negative controls transfected AsPC‐1 cells

The above finding that PWAR6 could negatively regulate YAP1 expression promoted us to assume that PWAR6 could also affect the target genes of Hippo‐YAP1 pathway, then contributed to the PDAC tumorigenesis. As expected, we analysed the data of the PDAC patient cohort collected in the GEPIA database; as shown in Figure S2A, PWAR6 expression negatively correlated with the levels of some Hippo‐YAP1 downstream genes (BIRC5, CCNB1, CCNE1, CDC20 and FOXM1) in PDAC specimens. qRT‐PCR assay further confirmed that the regulation of PWAR6 could significantly influenced the mRNA expression of these genes (Figure S2B). Next, to ascertain YAP1 was involved in PWAR6‐mediated tumour‐suppression role in PDAC, we performed a series of rescue experiments in AsPC‐1 and BxPC‐3 cells. As illustrated in Figure 6A‐C, the CCK‐8, BrdU and colony formation assays demonstrated that restoration of YAP1 partially rescued PWAR6‐mediated inhibitory effects on PDAC cell proliferation. The transwell invasion and migration assays revealed that overexpression of YAP1 recovered the inhibition of cell migration ability induced by PWAR6 overexpression (Figure 6D,E). Flow cytometry analysis revealed that YAP1 overexpression counteracted G0/G1 phase arrest induced by PWAR6 (Figure 6F, left panel). On the contrary, YAP1 knockdown restored cell cycle promoted by PWAR6 siRNA (Figure 6F, right panel). Furthermore, the apoptosis analysis showed that cell apoptosis enhanced by PWAR6 was significantly opposed by YAP1 co‐transfection (Figure 6G). The expression level of YAP1 was confirmed in Figure 6H as transfected with above indicated plasmids. These data suggest that YAP1 is a critical target gene of PWAR6 involved in PDAC tumorigenesis.

## 4 DISCUSSION

Large amounts of cancer research used to focus on protein‐coding genes that with potential to act as clinical biomarkers. However, <2% of the human genome is subsequently translated and the majority of the genomes in fact transcribed into ncRNAs.^18^ The characteristic of lncRNAs that do not encode proteins do not mean such RNAs do not have function. Accumulating evidence has revealed that lncRNAs vitally participate in cellular processes and a variety of human diseases, especially cancer.^19^ For example, our previous study reported that LINC00958 exerted oncogenic function in human head and neck squamous carcinoma.^20^ Moreover, Wang et al found that lncRNA GAS5‐AS1 suppressed cervical cancer cell tumorigenicity and metastasis by decreasing GAS5 N6‐methyladenosine (m6A) modification.^21^ In the present study, for the first time, we demonstrated that lncRNA PWAR6 was down‐regulated in PDAC tissues and cell lines. The biological function of PWAR6 in regulating malignant phenotypes of PDAC was also evaluated. Overexpression of PWAR6 inhibited cell proliferation and migration, induced cell G1/G0 phase arrest and promoted cell apoptosis. In contrast, depletion of PWAR6 contributed to the opposite effects. These data indicate that PWAR6 may act as an anti‐oncogene and might exhibit critical biological functions in the development of PDAC.

During tumorigenesis, the up‐ or down‐regulation of specific lncRNAs frequently occurs. In recent years, more and more studies have reported that dysregulated expression of lncRNAs in human cancers reflects the spectrum of tumour progression and could effectively forecast patient clinical outcomes.^22^ For instance, elevated expression of lncRNA 91H was found to be a predictor for shorter overall survival in hepatocellular carcinoma patients.^23^ Yan et al demonstrated that LINC00470 was frequently up‐regulated in gastric cancer tissues and cell lines. Overexpression of LINC00470 was correlated with TNM stage and distant metastasis status of gastric cancer.^24^ LncRNA CASC15 was verified to function as an unfavourable predictor for ovarian cancer. Low expression of CASC15 was closely linked to advanced TNM stage, poorer differentiation, and larger tumour size.^25^ Of note, many lncRNAs, such as HOTTIP, XIST, HULC, ROR and MALAT‐1, were reported to be associated with clinical characteristics of PDAC patients.^26^, ^27^ Our study indicated that lower level of PWAR6 is associated with higher TNM stage and distant metastasis status in PDAC patients. Furthermore, we demonstrated that higher PWAR6 expression correlates with better clinical outcome in patients. The data retrieved from KM plotter website further confirmed our results. Therefore, lncRNA PWAR6 serves as a promising biomarker for PDAC patients.

The above results suggested the tissue specificity and the tumour suppressor role of PWAR6 in PDAC. However, the genes that were affected by PWAR6 remains undocumented in PDAC. We thus screened a bunch of PWAR6‐related genes, among which YAP1 caught our attention. YAP1 is a transcriptional coactivator in Hippo pathway and considered to be encoded by a proto‐oncogene.^28^ By mediating its target gene transcription in most cancer cells, YAP1 plays multiple regulatory roles in cancer progression.^29^ In PDAC, overexpression of YAP1 correlated with liver metastasis and poor prognosis.^30^ The bioinformatic analysis using GEPIA server indicated a negative correlation trend between PWAR6 and YAP1. We further tested the expression pattern of YAP1 in PDAC tissues and adjacent pancreatic tissues by performing qRT‐PCR assay. It was evident that YAP1 was up‐regulated in PDAC tissues, and the Pearson analysis further reinforced the inverse correlation between PWAR6 and YAP1, which was also validated in our western blot and qRT‐PCR assays as PWAR6 suppressed the expression of YAP1. As to the mechanism of the regulation of YAP1 by PWAR6 remains unclear, which arouse our interest to explore.

Given that the subcellular location of lncRNAs may decide its functionality, we first analysed the distribution and subcellular location of PWAR6 in PDAC cells. The subcellular localization patterns of lncRNAs and their biological functions are closely related. In the cytoplasmic, lncRNAs target mRNA transcripts and modulate its stability and translation, whereas the nuclear lncRNAs are important epigenetic modulators of nuclear functions.^31^ The subcellular fractionation assay demonstrated that PWAR6 primarily localizes within the nucleus of PDAC cells. In the nucleus, many lncRNAs act by influencing the epigenetic status of protein‐coding genes through direct interactions with chromatin‐modifying factors.^32^ PRC2, one of the most studied chromatin‐modifying factors, is a histone methyltransferase required for epigenetic silencing during cancer development.^33^ The binding probability between PWAR6 and PRC2 was analysed by RNA‐protein interaction prediction (http://pridb.gdcb.iastate.edu/RPISeq/). Both the RF and SVM scores were higher than 0.5, indicating a high possibility for PWAR6 and PRC2 physical interaction. The methyltransferase EZH2 is the core subunit of the PRC2 complex, and previous studies have shown that numerous lncRNAs can bind to EZH2.^34^ For instance, lncRNA UCA1 mediates the expression of p21 and SPRY1 through interacting with EZH2, thus facilitating tumour progression of gastric cancer.^35^ LINC01133 inhibits breast cancer invasion and metastasis through repressing SOX4 expression by recruiting EZH2 to SOX4 promoter.^36^ In our present study, through RIP and RNA pull‐down assays, we identified direct interaction between PWAR6 and EZH2. ChIP assay using EZH2 and H3K27me3 antibodies demonstrated that EZH2 can directly bind to YAP1 promoter region and induce trimethylation of H3K27 in PDAC cells. Therefore, we speculated that PWAR6 exerts its tumour suppressive role by negatively regulating YAP1. As expected, PWAR6‐induced cell proliferation inhibition, cell cycle arrest and cell apoptosis could be partially reversed by YAP1 co‐transfection.

In conclusion, our study provided evidence for the first time that PWAR6 exerts tumour suppressor activity in human PDAC cells by interacting with EZH2 and facilitating its repression of YAP1, thus mediating proliferation, apoptosis and metastasis of PDAC. These findings indicate that PWAR6 is a critical molecule for PDAC tumorigenesis and could be a promising target for novel intervention and/or therapeutics in PDAC.

## CONFLICT OF INTEREST

The authors declare that there is no conflict of interest in this study.

## AUTHOR CONTRIBUTION

**Shanshan Huang:** Conceptualization (equal); Investigation (equal); Writing‐original draft (equal); Writing‐review & editing (equal). **Yaqi Li:** Data curation (equal); Investigation (equal). **Jinhua Hu:** Investigation (equal); Validation (equal). **Li Li:** Formal analysis (equal); Software (equal); Validation (equal). **Zhen Liu:** Data curation (equal); Methodology (equal); Validation (equal). **Hui Guo:** Formal analysis (equal); Project administration (equal); Software (equal). **Bailing Jiang:** Data curation (equal); Software (equal). **Jun Chen:** Project administration (equal). **Junhe Li:** Conceptualization (equal); Data curation (equal); Investigation (equal); Supervision (equal). **Xiaojun Xiang:** Conceptualization (equal); Funding acquisition (equal); Investigation (equal). **jun deng:** Conceptualization (equal); Funding acquisition (equal); Supervision (equal); Writing‐review & editing (equal). **Jianping Xiong:** Conceptualization (equal); Funding acquisition (equal); Supervision (equal); Writing‐review & editing (equal).

## Supporting information

Fig S1

Fig S2

## Data Availability

The data that support the findings of this study are available from the corresponding author upon reasonable request.
