# Supplementary figures and images for: LncRNA PWAR6 regulates proliferation and migration by epigenetically silencing YAP1 in tumorigenesis of pancreatic ductal adenocarcinoma
Source: J Cell Mol Med. 2021 Apr 8;25(9):4275–86. doi: 10.1111/jcmm.16480 (PMC8093982; doi:10.1111/jcmm.16480)

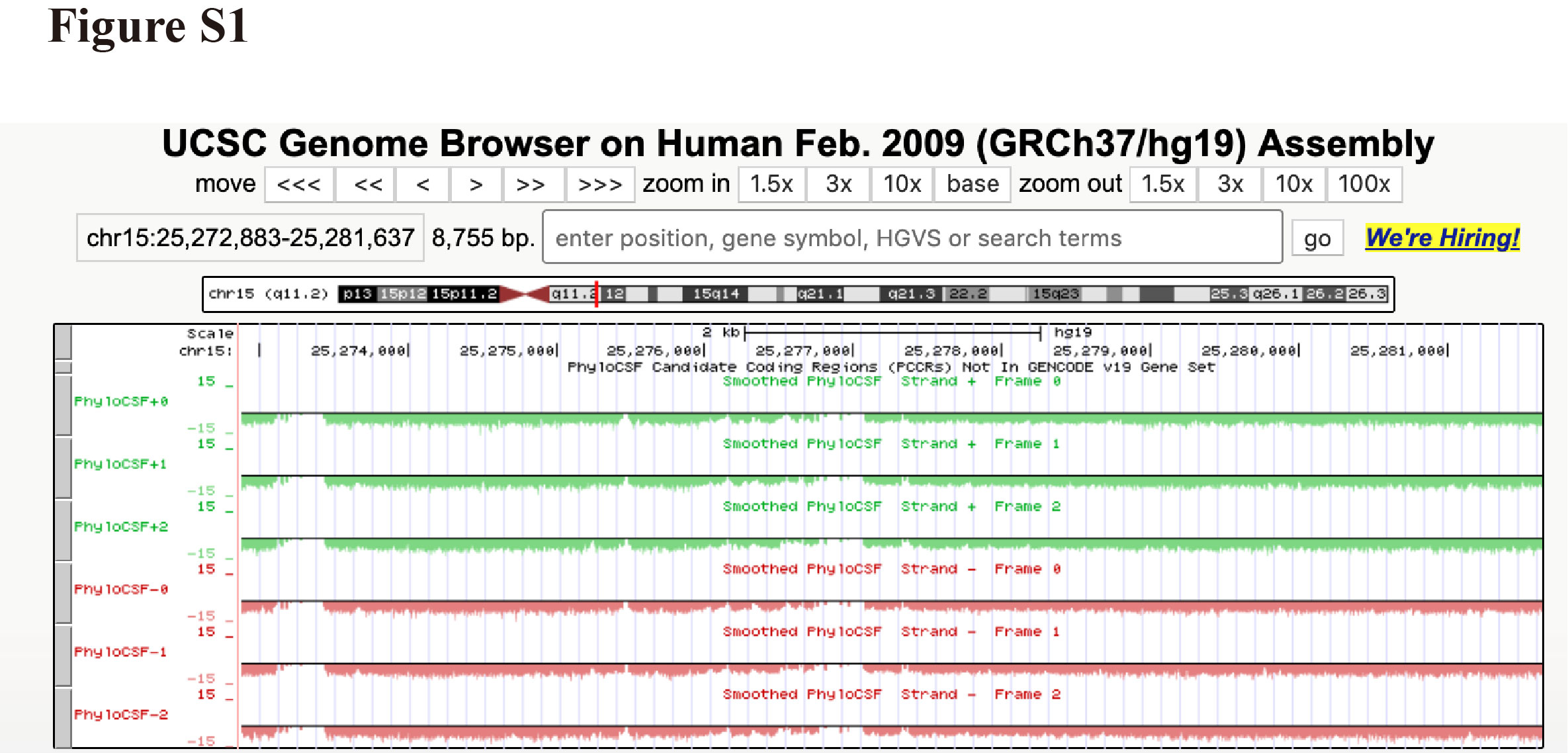

Supplement: Supplementary file 1 — Fig S1 [file JCMM-25-4275-s002.jpg]

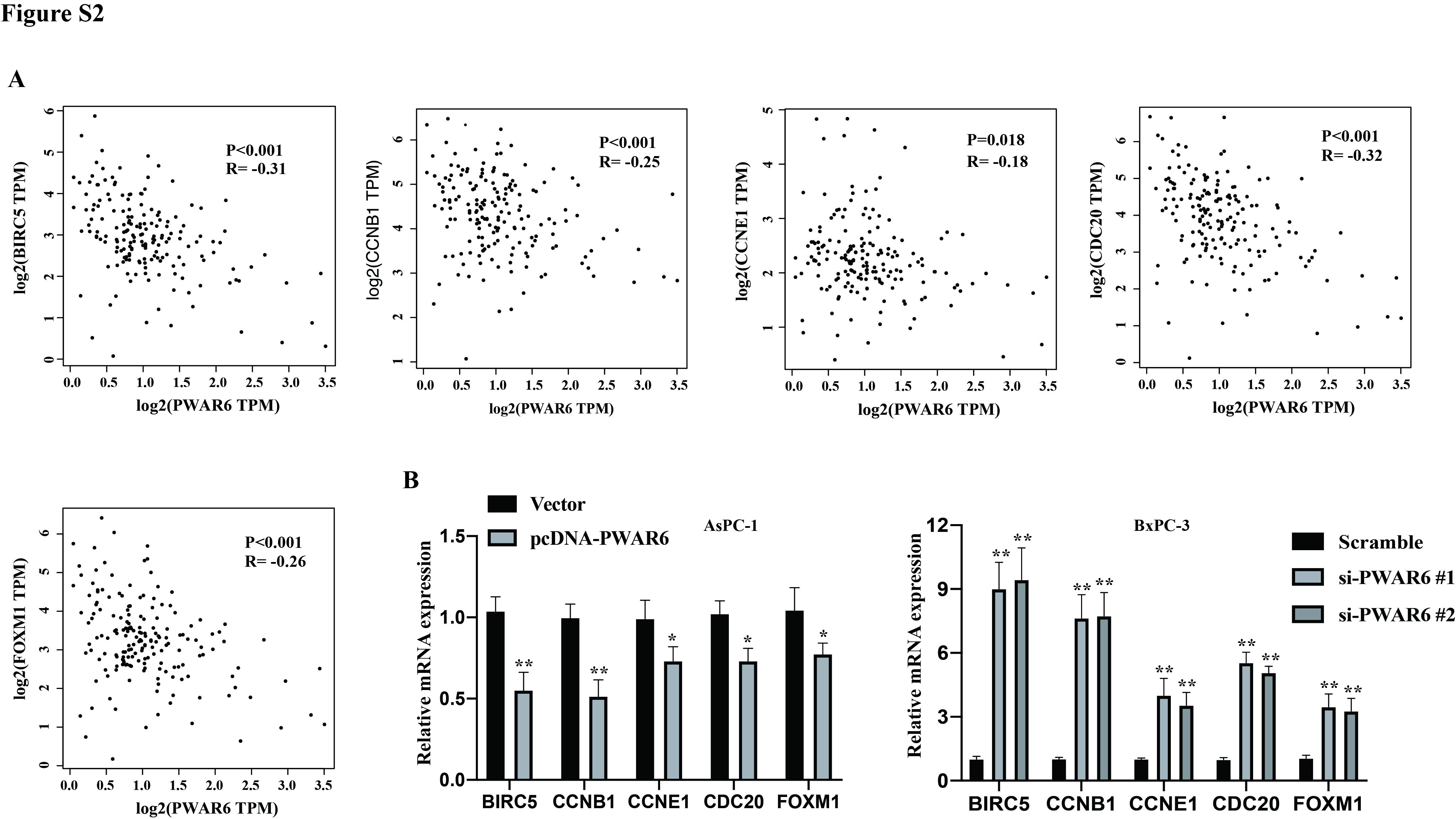

Supplement: Supplementary file 2 — Fig S2 [file JCMM-25-4275-s001.jpg]
